# Arthroscopically occult meniscotibial instability persists despite systematic transcondylar assessment: Incidence and associated factors during ACL reconstruction

**DOI:** 10.1051/sicotj/2026067

**Published:** 2026-09-23

**Authors:** Tsuneari Takahashi, Takumi Matsumoto

**Affiliations:** 1 Department of Orthopedics, Jichi Medical University Shimotsuke Japan; 2 Department of Orthopaedic Surgery, Ishibashi General Hospital Shimotsuke Japan

**Keywords:** ACL reconstruction, Meniscotibial instability, Ramp lesion, Vascularity, Probing

## Abstract

*Introduction*: Medial meniscus ramp lesions are recognized contributors to knee instability in anterior cruciate ligament (ACL)–injured knees. However, some knees may demonstrate functional instability at the meniscotibial attachment without visible structural disruption on arthroscopic inspection. The purpose of this study was to determine the incidence of probe-detected meniscotibial instability in knees without a visible ramp lesion on transcondylar inspection and to identify associated factors. *Methods*: Patients who underwent primary ACL reconstruction between January 2021 and January 2026 were retrospectively reviewed. The ramp region was evaluated using a standardized transcondylar view, followed by systematic probing. Meniscotibial instability was defined as abnormal mobility of the posterior horn of the medial meniscus at the meniscotibial attachment in the absence of visible structural disruption. Multivariable logistic regression and receiver operating characteristic (ROC) analyses were performed. *Results*: Of 171 patients, 113 met eligibility criteria. Forty-three had visible ramp lesions, leaving 70 for analysis. Meniscotibial instability was identified in 29 of 70 patients (41.4%). Preoperative side-to-side difference (SSD) in anterior tibial translation (OR, 1.81; *P* = 0.0024) and reduced ramp vascularity (OR, 3.36; *P* = 0.047) were independently associated with instability. An SSD cutoff of 5.0 mm showed acceptable discrimination (AUC, 0.734). *Conclusion*: Meniscotibial instability may be present despite normal arthroscopic appearance of the ramp region and is associated with increased preoperative instability. Systematic probing is essential to identify this occult functional abnormality.

## Introduction

Medial meniscus ramp lesions are frequently associated with anterior cruciate ligament (ACL) injuries and have been increasingly recognized as an important contributor to residual knee instability after ACL reconstruction [1, 2]. Accumulating biomechanical and clinical evidence suggests that untreated ramp lesions may compromise knee kinematics, increase strain on the reconstructed graft, and adversely affect meniscal integrity [2, 3]. Accordingly, accurate identification of ramp lesions during ACL reconstruction is strongly recommended, as routine anterior arthroscopic inspection may fail to detect clinically relevant pathology [4, 5].

Despite growing awareness, ramp lesions remain one of the most frequently missed meniscal pathologies during arthroscopy. Visualization of the posteromedial aspect of the medial meniscus posterior horn is technically challenging using standard anterior portals alone. To address this limitation, evaluation of the ramp region through the intercondylar notch using a transcondylar view from the anterolateral portal has been widely adopted as a practical method for arthroscopic assessment of the medial meniscus posterior horn [5].

However, even when the ramp region is systematically assessed using a transcondylar view, not all clinically relevant ramp lesions are readily apparent. In particular, tibial-side ramp lesions involving the meniscotibial attachment may not demonstrate obvious meniscocapsular separation on visual inspection alone. These lesions may appear stable or intact on arthroscopic visualization, yet demonstrate clear pathological mobility when assessed by systematic probing [1, 5]. Such arthroscopically inconspicuous tibial-side ramp lesions may therefore represent a persistent diagnostic blind spot, independent of the arthroscopic viewing approach. These lesions may therefore be overlooked not because of inadequate visualization, but because of their intrinsic pathological characteristics at the meniscotibial attachment.

While previous studies have reported the overall prevalence and risk factors of ramp lesions, most have focused on visually apparent lesions or magnetic resonance imaging (MRI)-based detection [1, 4]. The incidence of tibial-side ramp lesions detected only by probing, despite systematic arthroscopic evaluation of the ramp region using a transcondylar view, remains poorly defined. Furthermore, little is known about the preoperative and intraoperative factors that may predict the presence of these hidden yet potentially unstable lesions.

Given the established clinical importance of ramp lesions and the unacceptable consequences of missing them, a more refined understanding of tibial-side ramp pathology is required. Identifying patients at risk for probe-detected tibial-side lesions may help optimize intraoperative assessment strategies and prevent residual instability after ACL reconstruction.

Experimental studies have suggested that medial meniscus ramp pathology may progress biologically even when immediate mechanical instability is not apparent, highlighting the need for refined intraoperative assessment strategies [6, 7]. The purpose of this study was therefore to determine the incidence of tibial-side medial meniscus ramp lesions detected by probing in knees undergoing ACL reconstruction in which no obvious ramp lesion was identified on routine arthroscopic inspection using a transcondylar view. A secondary aim was to identify factors associated with the presence of these probe-detected tibial-side lesions.

## Methods

### Arthroscopic assessment and definition of tibial-side ramp lesions

#### Study population

This retrospective study included all consecutive patients who underwent primary ACL reconstruction between January 2021 and January 2026 at our affiliated hospital. ACL injury was diagnosed based on clinical examination and MRI. Patients who underwent revision ACL reconstruction or concomitant reconstruction of other major knee ligaments were excluded. All procedures were performed or supervised by experienced knee surgeons using a standardized arthroscopic protocol. The time from injury to surgery was recorded from medical charts. Chronic injury was defined as a time interval of ≥12 months from injury to surgery.

#### Arthroscopic evaluation

After routine diagnostic arthroscopy through standard anterolateral and anteromedial portals, the posterior horn of the medial meniscus was assessed. The ramp region was evaluated through the intercondylar notch using a transcondylar view from the anterolateral portal. The posterior meniscocapsular junction and the meniscotibial attachment were inspected under direct visualization. Cases in which a clearly visible meniscocapsular ramp lesion was identified were documented separately. The present analysis focused on knees in which no obvious ramp lesion was identified on visual inspection, despite systematic arthroscopic evaluation of the ramp region using a transcondylar view.

#### Arthroscopic classification of medial meniscus ramp lesions

Medial meniscus ramp lesions have been arthroscopically classified according to the involved structure, including injuries to the meniscocapsular junction, the meniscotibial attachment, or both [1, 5].

Meniscocapsular ramp lesions are typically characterized by visible separation between the posterior horn of the medial meniscus and the joint capsule, whereas tibial-side (meniscotibial) ramp lesions may appear arthroscopically intact on visual inspection alone and require probing for detection [1, 5]. The present study specifically focused on this tibial-side subtype.

#### Probing assessment

Following visual inspection, systematic probing of the posterior horn of the medial meniscus was performed in all cases. Probing was used to assess stability of both the meniscocapsular and meniscotibial attachments, as visual inspection alone may fail to detect clinically relevant ramp pathology [1, 5].

Meniscotibial instability was defined as functional abnormal mobility of the posterior horn of the medial meniscus at the meniscotibial attachment in the absence of visible structural disruption.

Instability was assessed using a standardized probing technique. The probe was applied to the inferior aspect of the posterior horn, and abnormal findings were defined as:

(1). lift-off of the posterior horn from the tibial plateau,
(2). formation of a meniscotibial gap, or
(3). excessive posterior translation compared with expected physiological mobility.

**Figure 1:**
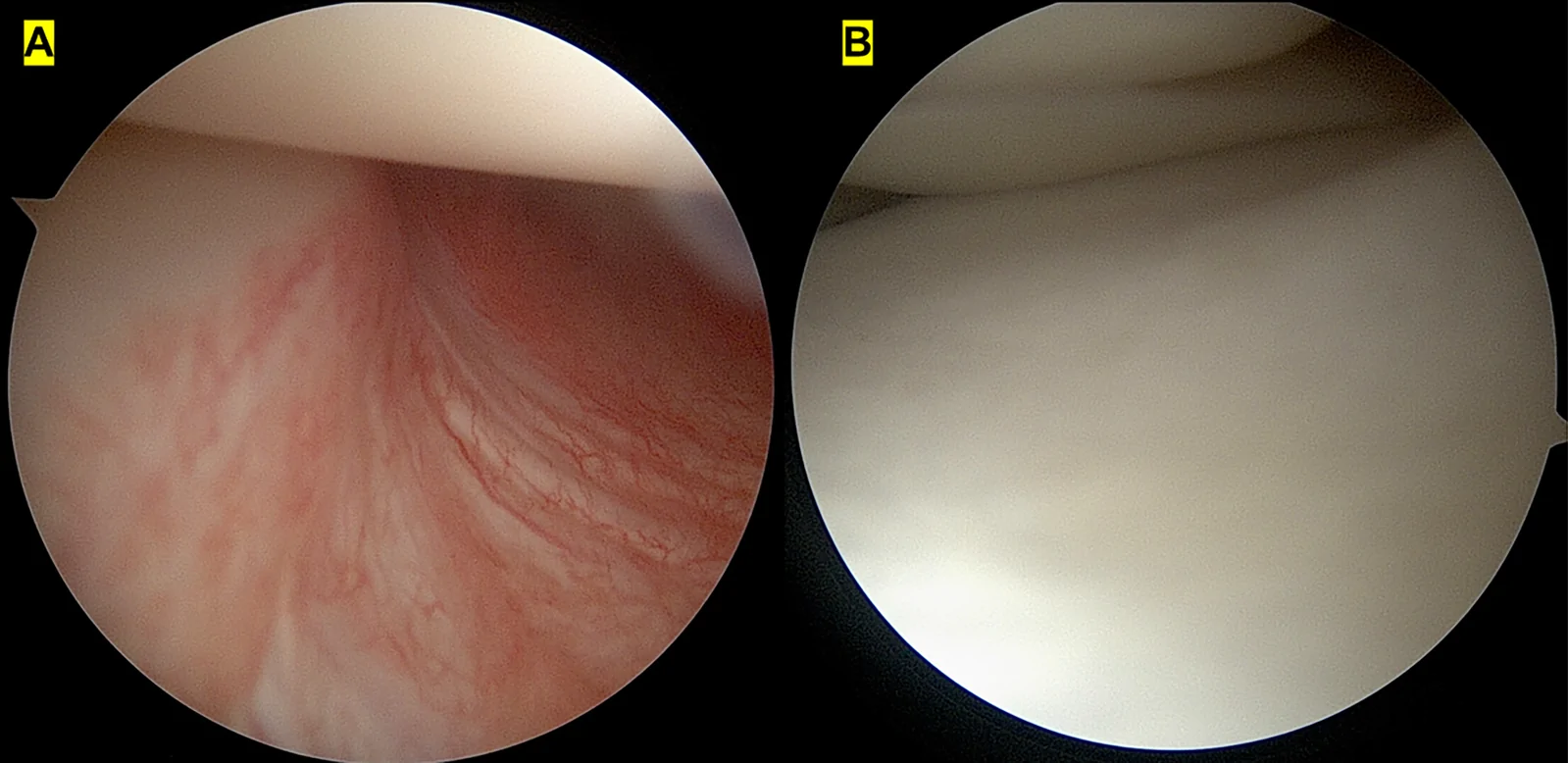
Arthroscopic appearance of a meniscotibial instability (left knee). Arthroscopic images of a left knee obtained from the anterolateral portal using a transcondylar view. (A) On visual inspection, no obvious meniscocapsular separation is observed at the posteromedial meniscocapsular junction. (B) Despite the inconspicuous appearance, pathological instability at the meniscotibial attachment is confirmed on probing, demonstrating abnormal mobility of the posterior horn with lift-off from the tibial plateau and formation of a meniscotibial gap. These findings are consistent with meniscotibial instability.

These findings were interpreted as functional instability rather than structural disruption (Figure 1).

#### Assessment of vascularity in the ramp region

Vascularity of the ramp region was assessed arthroscopically using the transcondylar view. The peripheral aspect of the posterior horn of the medial meniscus and the adjacent synovium were evaluated for visible synovial vessels and hyperemic changes.

The rationale for vascular assessment is supported by classical anatomical studies demonstrating that meniscal blood supply is limited to the peripheral portion of the meniscus and shows regional heterogeneity [8, 9].

Given that biological healing potential is closely related to local vascularity, assessment of the vascular environment in the ramp region may provide clinically relevant information for tibial-side ramp pathology. No intentional rasping, abrasion, or provocative maneuver was performed to induce bleeding, in order to avoid iatrogenic alteration of the native biological environment.

**Figure 2:**
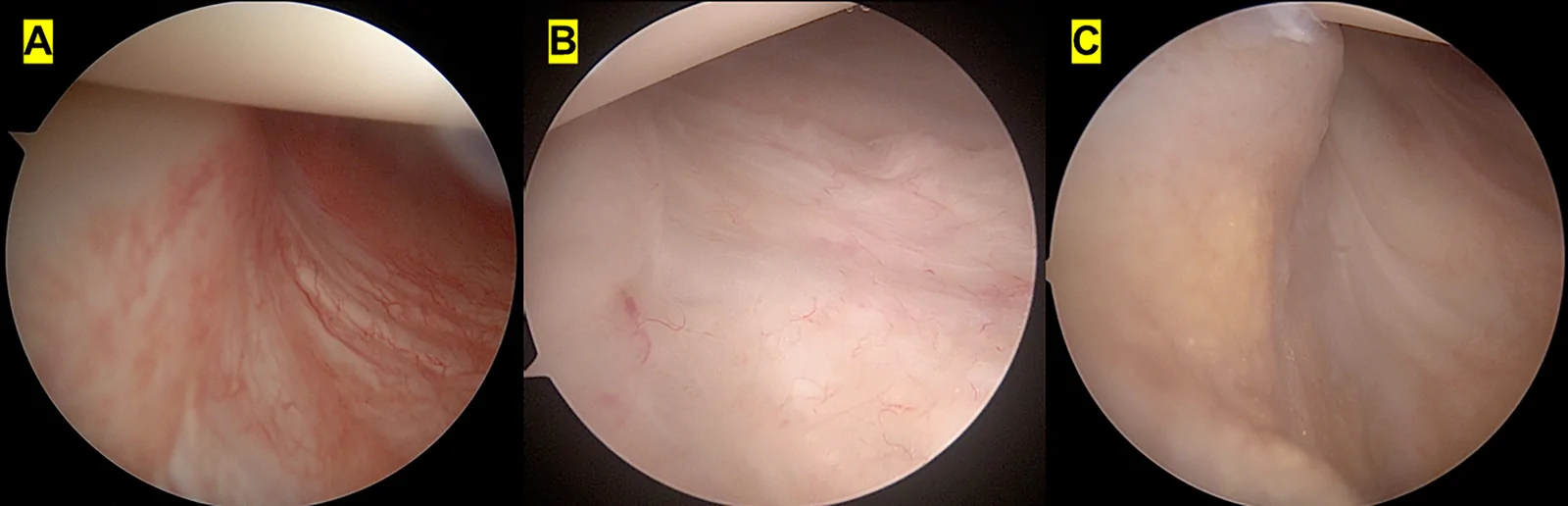
Qualitative arthroscopic assessment of ramp-region vascularity (left knee). Arthroscopic transcondylar-view images of the ramp region in a left knee, obtained from the anterolateral portal. Ramp-region vascularity was qualitatively graded based on the arthroscopic appearance of synovial vessels and hyperemia adjacent to the posteromedial meniscus. (A) Adequate vascularity, showing clearly visible synovial vessels and marked hyperemic changes. (B) Reduced vascularity, showing sparse or poorly visualized vessels with minimal hyperemia. (C) Poor vascularity, showing absence of visible vessels and hyperemia. Vascularity worsens from A to C.

Based on arthroscopic appearance alone, vascularity was categorized qualitatively as:

- Adequate: clearly visible synovial vessels and/or marked hyperemia adjacent to the ramp region,
- Reduced: sparse or poorly visualized vessels with minimal hyperemic changes, or
- Poor: absence of visible vessels and hyperemia (Figure 2).

Vascularity was assessed before probing to avoid any influence of arthroscopic manipulation on the appearance of synovial vessels and hyperemia. This qualitative classification was used to reflect potential differences in biological healing capacity at the peripheral posterior horn of the medial meniscus, based on established anatomical evidence of regional variation in meniscal vascularity. Because a validated quantitative arthroscopic grading system for meniscal vascularity has not been established, vascularity was assessed qualitatively in the present study [8, 9].

#### Data recording

The presence of meniscotibial instability was recorded intraoperatively. Arthroscopic images or videos were obtained when feasible. All assessments followed the same predefined protocol throughout the study period.

### Statistical analysis

The primary outcome was the presence of meniscotibial instability. Independent variables were selected a priori based on previously reported associations with ramp lesions and meniscal healing potential [1, 3].

Univariate analyses were performed, followed by multivariable logistic regression analysis to identify independent factors associated with the presence of meniscotibial instability. Because only a small number of knees demonstrated poor vascularity, the reduced and poor vascularity categories were prespecified as a single “not adequate” group for logistic regression analysis. Odds ratios (ORs) with 95% confidence intervals (CIs) were calculated. Independent variables included demographic factors and preoperative clinical variables, as well as arthroscopic ramp vascularity (adequate vs not adequate) as a candidate biological predictor.

To evaluate the discriminatory performance of significant predictors, receiver operating characteristic (ROC) curve analysis was performed, and the optimal cutoff value was determined. Model discrimination was assessed using the area under the ROC curve (AUC). Statistical significance was set at *P* < 0.05.

## Results

### Patient enrollment and study cohort

**Figure 3:**
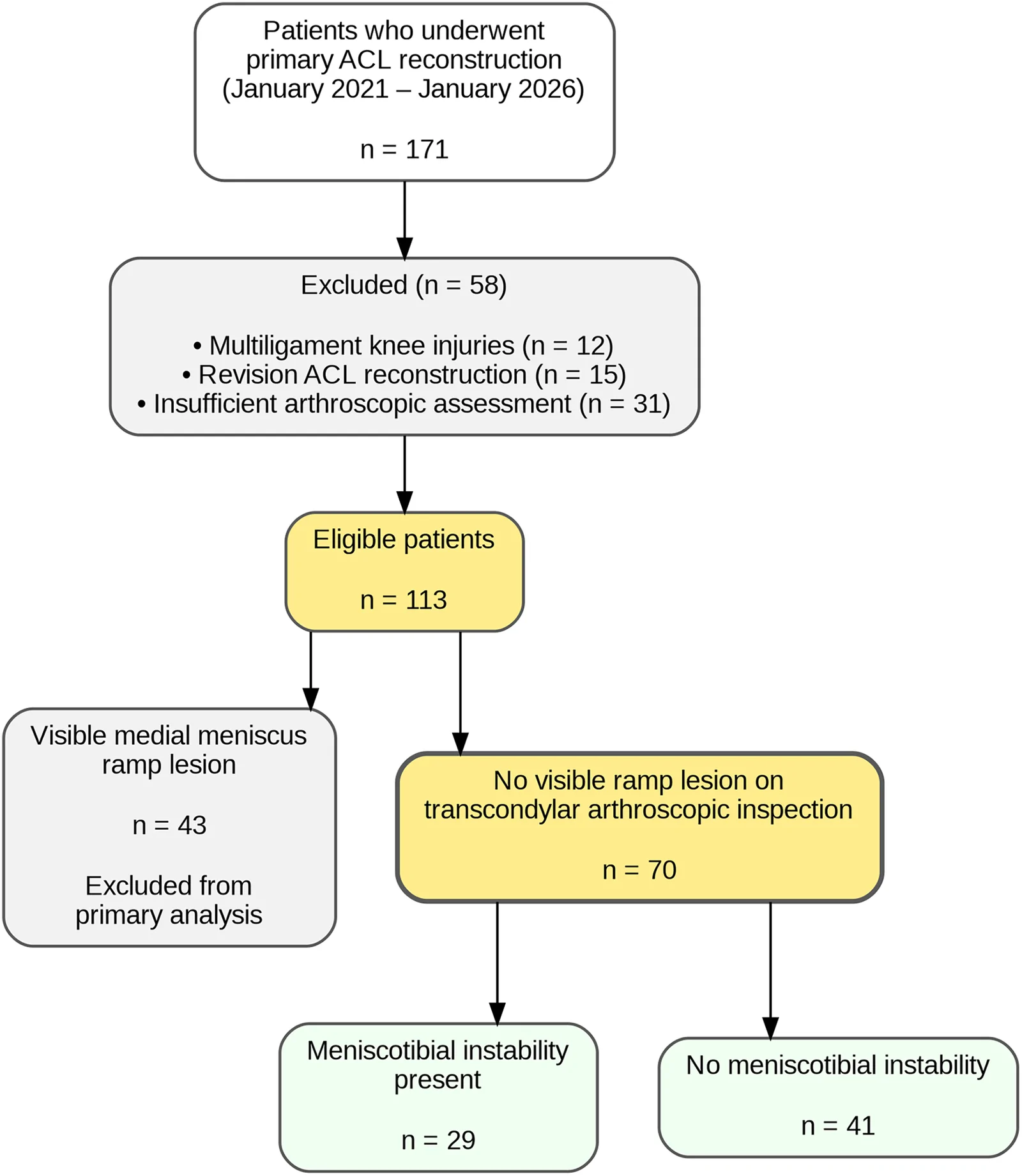
Patient flow diagram. Flow diagram illustrating patient selection and study cohort formation. Among 171 consecutive patients who underwent primary ACL reconstruction, 58 were excluded because of multiligament knee injury (*n* = 12), revision ACL reconstruction (*n* = 15), or insufficient arthroscopic assessment (*n* = 31). Of the remaining 113 eligible patients, 43 had a visible medial meniscus ramp lesion identified during arthroscopic inspection and were excluded from the primary analysis. The final study cohort comprised 70 patients without a visible ramp lesion on transcondylar arthroscopic assessment, among whom meniscotibial instability was identified in 29 patients.

A total of 171 patients who underwent primary ACL reconstruction were initially enrolled. After excluding patients with multiligament knee injuries (12 knees), revision ACL reconstruction (15 knees), and insufficient arthroscopic assessment (31 knees), 113 patients were eligible for analysis (Figure 3).

Among these, 43 patients (38.1%) had an arthroscopically visible medial meniscus ramp lesion. Therefore, the remaining 70 patients (61.9%) without an obvious ramp lesion on visual inspection were included in the final cohort for evaluation of meniscotibial instability. Meniscotibial instability was identified in 29 of 70 patients (41.4%). Patient demographics were shown in Table 1.

**Table 1:** Patient demographics and preoperative characteristics (*N* = 70).

Variable	Value
Age (years)	41.4 ± 16.6
Sex (female), *n* (%)	25 (35.7%)
Side (Right), *n* (%)	43 (61.4%)
Height (m)	1.64 ± 0.09
Weight (kg)	62.7 ± 13.0
BMI (kg/m^2^)	23.1 ± 3.7
Chronic injury, *n* (%)	28 (40.0%)
Preoperative lysholm score, median (IQR)	60 (45–78)
Preoperative tegner activity scale, median (IQR)	5 (3–6)
Preoperative IKDC grade, *n* (%)	
Grade B	2 (2.9%)
Grade C	32 (45.7%)
Grade D	35 (50.0%)
Missing	1 (1.4%)
Preoperative SSD (mm)	5.1 ± 2.2
Preoperative pivot-shift grade, *n* (%)	
Negative	9 (12.9%)
Glide	29 (41.4%)
Clunk	27 (38.6%)
Gross	5 (7.1%)
Ramp vascularity (adequate), *n* (%)	32 (45.7%)
Ramp vascularity (reduced/poor), *n* (%)	38 (54.3%)

BMI: body mass index, IKDC: international knee documentation committee, SSD: side-to-side difference.

### Logistic regression and ROC curve analysis

Multivariable logistic regression analysis demonstrated that preoperative SSD in anterior tibial translation was significantly associated with meniscotibial instability (odds ratio [OR], 1.81; 95% CI, 1.23–2.66; *P* = 0.0024). In addition, reduced ramp vascularity (vascularity not adequate) was also independently associated with meniscotibial instability (OR, 3.36; 95% CI, 1.02–11.10; *P* = 0.047). Because only a small number of patients were classified as having poor vascularity, the reduced and poor vascularity groups were combined into a single “not adequate” category for multivariable analysis.

**Figure 4:**
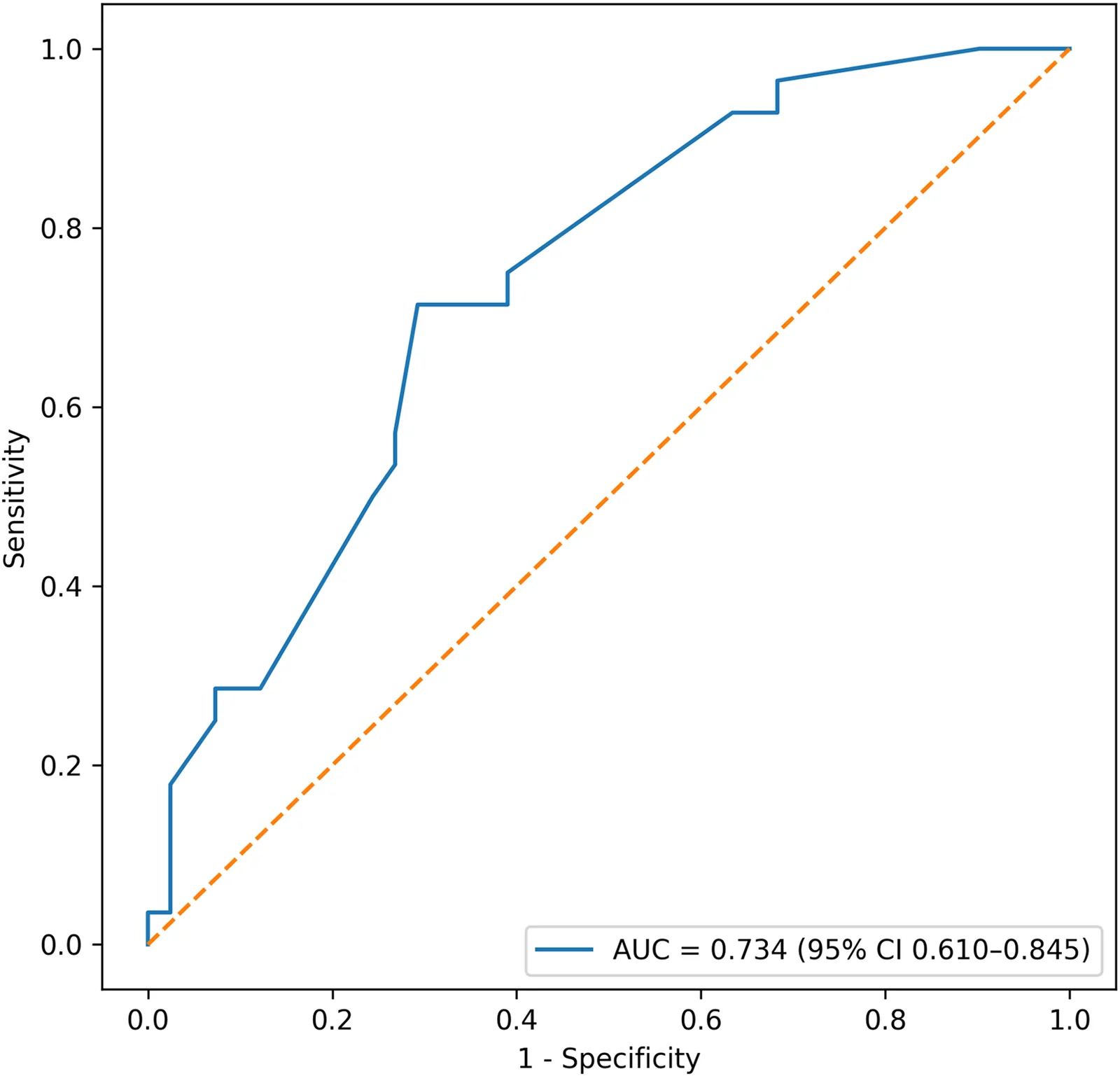
Receiver operating characteristic (ROC) curve demonstrating the discriminatory performance of preoperative side-to-side difference (SSD) in anterior tibial translation for predicting meniscotibial instability in patients without an obvious ramp lesion on transcondylar arthroscopic inspection. The area under the curve (AUC) was 0.734 (95% confidence interval, 0.618–0.851). An SSD cutoff value of 5.0 mm was identified as the optimal threshold for discrimination. The dashed diagonal line indicates the reference line of no discrimination.

ROC curve analysis identified an optimal SSD cutoff value of 5.0 mm for predicting meniscotibial instability. The AUC was 0.734 (95% CI, 0.618–0.851), indicating acceptable discriminatory performance (Figure 4).

## Discussion

### Mechanical determinants of hidden tibial-side ramp lesions

The most important finding of the present study was that meniscotibial instability was frequently detected only by probing, despite systematic transcondylar arthroscopic evaluation. This supports previous literature emphasizing that visual inspection alone may fail to identify clinically relevant ramp pathology and that systematic assessment strategies are required to reduce missed lesions [5, 10].

The present study does not attempt to redefine established structural classifications of medial meniscus ramp lesions; rather, it describes a subgroup of knees that demonstrate functional instability at the meniscotibial attachment in the absence of visible structural disruption. This distinction is critical. Unlike previously described ramp lesions involving definite meniscocapsular or meniscotibial tears, the current findings represent a functional arthroscopic finding rather than a structural diagnosis.

Whether meniscotibial instability represents an early stage of structural failure, a distinct pathological entity, or the upper end of physiological variation remains unknown. Therefore, we do not propose that meniscotibial instability should replace existing structural classifications of ramp lesions. Rather, we believe that it represents a clinically relevant functional finding that may coexist with structurally intact meniscotibial attachments and warrants further validation through imaging, biomechanical, and histological studies.

Accordingly, the higher incidence observed in the present study, compared with previous reports, most likely reflects differences in diagnostic definitions rather than overdiagnosis.

From a mechanical standpoint, ramp lesions have been recognized as contributors to abnormal knee kinematics in ACL-deficient knees, potentially influencing anterior and rotational stability [2, 11].

In the present study, preoperative anterior instability (SSD) was independently associated with meniscotibial instability, supporting the role of mechanical stress in the development and persistence of these hidden lesions. Importantly, reduced ramp vascularity was also identified as an independent factor, indicating that biological vulnerability may contribute to lesion persistence and pathological mobility at the meniscotibial attachment. Taken together, these results support a combined mechanical–biological model of meniscotibial instability, in which mechanical stress acts on a biologically disadvantaged attachment, resulting in lesions that are arthroscopically inconspicuous yet unstable on probing.

These results provide a clinically relevant message: patients with greater preoperative anterior instability may harbor hidden meniscotibial instability, and systematic probing should be performed to avoid missing mechanically unstable lesions that are not apparent on visualization alone. Given that tibial-side ramp lesions can remain arthroscopically inconspicuous, probing-based assessment appears essential for accurate intraoperative diagnosis.

### Exploratory considerations on biological factors

In this study, reduced ramp vascularity was independently associated with meniscotibial instability in the multivariable analysis. However, this association should be interpreted with caution because the confidence interval was relatively wide and statistical significance was borderline (OR 3.36, 95% CI 1.02–11.10; *P* = 0.047). Therefore, the biological role of ramp-region vascularity remains exploratory and requires confirmation in larger prospective studies. Although probing-based assessment is inherently subjective, it remains an essential component of arthroscopic evaluation of the posterior horn. The present study aimed to standardize probing findings based on specific observable features, including lift-off, gap formation, and excessive mobility. Nevertheless, the absence of quantitative thresholds remains a limitation and warrants further investigation.

### Relationship to previous experimental findings

Our previous experimental studies provide important biological context for meniscotibial instability. Using a porcine ACL reconstruction model, we demonstrated that untreated medial meniscus ramp lesions did not significantly affect anterior knee laxity or graft structural properties under cyclic loading, yet resulted in clear histological deterioration of the medial meniscus [7].

Although the present study primarily highlights mechanical instability as the key determinant of hidden tibial-side ramp lesions, further investigations integrating clinical, mechanical, and biological data are warranted to better define which ramp lesions are most likely to persist and remain clinically relevant.

Reliability of this qualitative grading system was not formally evaluated and should be investigated in future studies.

### Limitations

This study has several limitations. First, its retrospective design may introduce selection bias, and causal relationships between preoperative instability and meniscotibial instability cannot be established. Second, although arthroscopic assessment was performed using a standardized transcondylar protocol, detection of meniscotibial instability depends on probing technique and surgeon interpretation, which may introduce measurement variability. Third, vascularity of the ramp region was assessed qualitatively based on arthroscopic appearance without inducing iatrogenic bleeding. Although reduced ramp vascularity was identified as an independent factor in the multivariable analysis, this association should be interpreted with caution because of the relatively wide confidence interval and borderline statistical significance. Therefore, findings related to vascularity should be considered exploratory, and the clinical relevance of arthroscopic vascular assessment remains uncertain. Interobserver and intraobserver reliability of the vascularity grading system were not assessed and therefore remain unknown. Accordingly, the reproducibility of this qualitative assessment should be confirmed in future prospective studies.

Finally, this study focused on intraoperative detection and associated factors of meniscotibial instability, and postoperative clinical outcomes were not evaluated. Additionally, the definition of meniscotibial instability is based on functional assessment rather than structural confirmation, and no gold-standard reference, such as histological or imaging validation, was available. Therefore, the findings should be interpreted as describing a functional abnormality rather than a confirmed structural lesion. Future prospective studies with longitudinal follow-up are required to clarify the clinical impact of these hidden lesions and the benefits of their treatment.

## Conclusion

Meniscotibial instability may be present despite systematic transcondylar arthroscopic visualization and often requires systematic probing to detect it. Preoperative anterior instability and reduced ramp vascularity were independently associated with meniscotibial instability, underscoring the importance of systematic probing during ACL reconstruction, particularly in patients with increased preoperative instability and biologically unfavorable ramp appearance. These findings support the concept that meniscotibial instability represents a distinct functional pathology that cannot be identified by visual inspection alone.

## Data Availability

The datasets used and/or analyzed during the current study are available from the corresponding author upon reasonable request.
